# Efficacy of Oral Vitamin A in Reducing β-hCG Levels in Low-Risk Gestational Trophoblastic Neoplasia Patients

**DOI:** 10.31557/APJCP.2020.21.11.3325

**Published:** 2020-11

**Authors:** Yudi Mulyana Hidayat, Eppy Darmadi A, Sylvia Rachmayati, Windy Puspa Kusumah, Tono Djuwantono, Akhmad Yogi Pramatirta, Dodi Suardi

**Affiliations:** *Department of Obstetrics and Gynecology, Faculty of Medicine, Universitas Padjadjaran/Dr. Hasan Sadikin General Hospital, Bandung, Indonesia. *

**Keywords:** β-hCG levels, gestational trophoblastic neoplasia, methotrexate chemotherapy, vitamin A

## Abstract

**Objective:**

Low-risk gestational trophoblastic neoplasia (GTN) is generally treated with single agent chemotherapy and methotrexate (MTX) as a first-line therapy. Vitamin A helps to increase trophoblast cell regression, as well as to decrease β-hCG levels. Vitamin A also increases the effectiveness of MTX by inducing more malignant cell death than MTX alone. Therefore, the aim of the current study was to analyze the changes in β-hCG levels in low-risk GTN patients following vitamin A administration.

**Methods:**

This study was a randomized clinical trial, which examined initial serum vitamin A and β-hCG levels in GTN patients before and after three cycles of MTX therapy. Patients were given vitamin A supplementation of 6,000 IU (1.8 mg RAEs) per day, and the changes in serum β-hCG were observed after three cycles. Patients were grouped by β-hCG levels (decreased or stagnant).

**Results:**

A total of 32 low-risks GTN patients were divided into the intervention group (16 patients who received vitamin A supplementation) and the control group (16 patients who did not receive vitamin A supplementation). In the intervention group, the average initial β-hCG level was 170,949.3 ± 354,452.1 mIU/mL, and the average β-hCG post-cycle level was 1,611.9 ± 3,652.5 mIU/mL. In the control group, the average initial β-hCG level was 178,834.1 ± 2913844.6 mIU/mL, and the average β-hCG post-cycle level was 25,388.5 ± 58,437.7 mIU/mL.

**Conclusion:**

In patients with low-risk GTN who underwent MTX chemotherapy, the levels of β-hCG and the incidence of chemo resistance in the intervention group were lower than those in the control group. Older age may also influence the incidence of chemo resistance in GTN patients. Oral administration of 6,000 IU vitamin A could help to reduce β-hCG levels in low-risk GTN patients who receive MTX chemotherapy.

## Introduction

Gestational trophoblast disease (GTD) comprises a group of diseases that develop after conception causes abnormal placental development characterized by abnormal trophoblastic cell proliferation. GTD includes hydatidiform mole, choriocarcinoma, placental-site trophoblastic tumor, epithelioid trophoblastic tumor, and gestational trophoblast neoplasia (GTN) (Garner et al., 2007). Gestational trophoblast tumors (GTTs) represent a form of malignant degeneration caused by continual abnormal trophoblastic cell proliferation (Niemann et al., 2015). The incidence of GTN in Indonesia is relatively high; the age of onset is commonly between the ages of 14–49 years, with an average of 31.2 years. The incidence of hydatidiform mole in Indonesia is estimated to be around 1:51–1:141 pregnancies, while in Bandung city, the estimated incidence is 1:427 pregnancies. Moreover, the incidence of GTTs is 1: 822 pregnancies (Garner et al., 2007).

Low-risk GTTs are generally treated with single agent chemotherapy and methotrexate (MTX) as a first-line therapy, which tends to show a good response in patients. Patients can be maintained through the acute phase, or treated more intensely if MTX resistance occurs (Niemann et al., 2015).

Vitamin A is a fat soluble vitamin which is absorbed through a carrier protein (Goncalves et al., 2015). Vitamin A has an important role in the regulation of cell proliferation, differentiation, and apoptosis by increasing p53 activity, which causes arrest in the G1 phase and the Bcl-2 gene which encourages apoptosis (Ghasemian et al., 2018). These roles of vitamin A are considered to have a synergistic effect on the action of MTX in inhibiting cell proliferation in the S phase of the cell cycle (Skubisz and Tong, 2012; Ghasemian et al., 2018). Low levels of vitamin A are thought to be a predisposing factor of trophoblastic cell proliferation in hydatidiform mole (Andrijono et al., 1997; Andrijono et al., 2007; Andrijono and Muhilal, 2010). Indeed, two case-control studies demonstrated that the risk of molar pregnancy progressively increased with reduced intake of animal fat and carotene, which are the precursors of vitamin A (Garner et al., 2007). In addition, two other studies showed that vitamin A helps to decrease serum β-hCG levels in patients with low-risk GTTs who receive MTX therapy (Sutanto et al., 2012; Ghasemian et al., 2018). Mouse studies on the absorption and storage of vitamin A after oral, subcutaneous, or intramuscular administration demonstrated that oral administration was the most effective in terms of liver storage. Subcutaneously injected vitamin A was approximately 35% as efficient as the same amount administered orally over a 5 day period, whereas intramuscular injection was only 2% as effective as the oral route. Therefore, in the current study, we used oral vitamin A, which is easily available in pharmacies, inexpensive, and easier to use. We sought to investigate the changes in β-hCG levels in patients with low-risk gestational trophoblastic neoplasia following vitamin A administration.

## Materials and Methods

*Design*

This study was a randomized clinical trial that examined initial serum vitamin A and β-hCG levels in GTN patients before and after MTX three cycle therapy. The patients were given vitamin A supplementation of 6,000 IU (1.8 mg RAEs) per day, and the changes in serum β-hCG were observed after three cycles. The patients were grouped by whether their β-hCG levels decreased or remained stagnant.

*Participants and recruitment*

The study subjects were patients who were diagnosed with low-risk GTT, and who were undergoing chemotherapy at RSUP Dr. Hasan Sadikin Hospital Bandung. Sample selection was made from a population of low-risk GTT patients who were undergoing chemotherapy at RSUP Dr. Hasan Sadikin Hospital Bandung using a randomized controlled trial sampling technique.

The inclusion criteria were patients with a diagnosis of low-risk GTT, who were planned to undergo, or who were undergoing the first cycle of single agent MTX chemotherapy, and who consented to participation in the study. The exclusion criteria were patients with a diagnosis of high-risk GTT, with poor adherence to chemotherapy and vitamin A therapy, and who were undergoing total hysterectomy.

The sample size was calculated using the following formula:

$n_{1}=n_{2}=2{\left(\frac{Z_{a}+Z_{\beta }}{X_{1}-X_{2}}\right)}^{2}$

The type I bias was limited to 5%, with a one-way hypothesis resulting in Zα = 1.64. The type II bias was limited to 20%, resulting in Zβ = 0,84.

$n_{1}=n_{2}=2{\left(\frac{{(Z}_{a}+Z_{\beta })S}{X_{1}-X_{2}}\right)}^{2}$

$n_{1}=n_{2}=2{\left(\frac{\left(1.64+0.84\right)\mathrm{*1}}{1}\right)}^{2}$

$=2\left(6.15\right)=12.3\approx 13$

Therefore, the minimum sample for this study was 13 subjects, which, when considering an excess of 10% to account for possible subject exclusion, resulted in a final minimum sample of 

13+1.3=14.3≈15

*Variables and measurements*

The independent variables of this study were vitamin A level and supplementation in GTT patients, while the dependent variable was β-hCG levels. 

Vitamin A levels were obtained from micro ELISA examination results from the Clinical Pathology Laboratory of Dr. Hasan Sadikin Hospital. β-hCG levels were obtained from patients’ medical records.

*Statistical analysis*

The normality of the numerical data distributions was assessed using the Shapiro-Wilk test before analysis. Comparison of the characteristics of the two research groups was performed using the unpaired t-test if the data were normally distributed and the Mann-Whitney test if the data were not. Categorical data were analyzed with the Chi-Square test; if the Chi-Square requirements were not met, then the Exact Fisher test was used for 2 x 2 Tables, and the Kolmogorov Smirnov test was used for all other tables. The Chi-Square requirement was that there was no expected value less than 5 in as much as 20% of the table. P-values ≤ 0.05 were considered statistically significant. The obtained data were recorded in a special form, and then processed through SPSS version 24.0 for Windows.

*Ethics approval*

Patients were informed of the procedure, risk, and benefit of the study, including vitamin A administration and MTX chemotherapy. Patients were also given information about voluntary participation and the right to refuse to participate in research. The patients agreed to be involved in this study, and will be given written informed consent to be signed. This study was approved by the Research Ethics Committee of the Faculty of Medicine, Padjadjaran University/Dr. Hasan Sadikin Hospital.

## Results

A total of 32 low-risk GTN were included, and divided into two groups, the intervention group (16 patients who received vitamin A supplementation) and the control group (16 patients who did not receive vitamin A supplementation). Two patients from the control group dropped out of the study.

The age, initial β-hCG levels, vitamin A levels, and Hb levels were compared between the intervention and control groups. In the intervention group, the average age of the patients was 32.3 years, the average initial β-hCG level was 170,949 mIU/mL and the average vitamin A level was 0.63. In the control group, the average age of the patients was 34.3 years, the average initial β-hCG level was 178,834 mIU/mL, and the average vitamin A level was 0.69. There were no significant differences between the intervention and control groups with regards to age, initial β-hCG levels, vitamin A levels, and Hb levels (p > 0.05).

The percentage change in third post-cycle β-hCG levels was compared between the intervention and control groups. In the intervention group, the average percentage change in third post-cycle β-hCG levels was −98.52 ± 2.64, while in the control group it was −77.97 ± 39.75, and there were no significant differences between the groups (p > 0.05, Mann-Whitney test).

In the intervention group, the average initial β-hCG level was 170,949.3 ± 354,452.1 mIU/mL, and the average post-cycle β-hCG level was 1,611.9 ± 3,652.5 mIU/mL. In the control group, the average initial β-hCG level was 178,834.1 ± 2913844.6 mIU/mL, and the average β-hCG post-cycle level was 25,388.5 ± 58,437.7 mIU/mL. The Wilcoxon test was used to obtain the p-value of the control and intervention groups, and demonstrated significant differences in the initial and post-cycle β-hCG levels in both the intervention and control groups (p < 0.001 and p < 0.013, respectively).

We next compared the achievement of remission and the occurrence of MTX resistance between the two groups (Table 4.). In the intervention group, patients who experienced remission achieved 16 (100.0%), and patients who experienced no resistance or 0.0%. In the control group, as many as 9 (64.3%) patients experienced remission, while as many as 5 (53.7%) experienced resistance. The statistical test with Exact Fisher was statistically significant. Patients in the intervention group have a lower percentage of resistance events compared to the control group. Based on the table 4. patients in remission group had an average age of 31 years, and an average initial β-hCG level of 182.807.6 ± 350.287.20. The patients in the resistant group had an average age of 43 years, and an average initial β-hCG level of 133,735.2 ± 96,696.7. Statistical test results on the Table 4. showed significant differences between the age of patients in the remission and resistant groups (p < 0.05), but no significant differences in the initial β-hCG level (p > 0.05).

**Table 1:** Comparison of Age, Initial β-hCG Levels and Initial Vitamin A Levels between the Intervention and Control Groups

Variable	Group		p-value
	Intervention n = 16	Control n = 14	
Age (years)			0.569
Mean (SD)	32.3 (8.5)	34.3 (10.3)	
Median	32.0	37.0	
Range (min–max)	19.0–46.0	19.0–48.0	
Initial β-hCG value			0.140
Mean (SD)	170.949.3 (354.452.1)	178.834.1 (291.844.6)	
Median	6.600.0	95.104.5	
Range (min–max)	50.0–1.256.000.0	387.2–1.125.000.0	
Vitamin A level			0.394
Mean (SD)	0.63 (0.40)	0.69 (0.39)	
Median	0.57	0.61	
Range (min–max)	0.25 – 1.83	0.29 – 1.77	
Hb level			0.922
Mean (SD)	11.9 (1.1)	11.8 (1.8)	
Median	12.1	11.7	
Range (min–max)	9.6–13.2	8.3 – 14.1	

For numerical data, the p-value was generated by an unpaired t-test if the data were normally distributed, and by the Mann-Whitney test if the data were not. *Indicates significance (p < 0.05).

**Table 2:** Comparison of Percentage Change in β-hCG Levels Post Third Cycle of Chemotherapy between the Intervention and Control Groups

Variable	Group		p-value
	Intervention n = 16	Control n = 14	

Percentage change in β-hCG levels post third cycle chemotherapy			0.190
Mean ± SD	-98.52 ± 2.64	-77.97 ± 39.75	
Median	-99.65	-99.14	
Range (min–max)	-99.999-(-90.95)	-99.996-10.339	

For numerical data, the p-value was generated by the Mann-Whitney test as the data were not normally distributed. *Indicates significance (p < 0.05).

**Table 3:** Comparison of Pre-Chemotherapy β-hCG Levels (initial) and Post Third Cycle Chemotherapy between the Intervention and Control Groups

Variable	β-hCG level		p-value
	pre-chemotherapy (initial)	Post third cycle chemotherapy	
Vitamin A supplementation			
Yes			
Mean ± SD	170.949,3 (354.452,1)	1.611,9 ± 3.652,5	<0,001*
Median	6.600,0	2,0	
Range (min–max)	50.0–1.256.000.0	1.3–10.690.0	
No			
Mean ± SD	178.834.1 (2913844.6)	25.388.5 ± 58.437.7	0,013*
Median	95.104.5	3.050.0	
Range (min–max)	387.2–1.125.000.0	0.4–163.200.0	

For numerical data, the p-value was generated by the Wilcoxon test as the data were not normally distributed. *Indicates significance (p < 0.05).

**Table 4:** Remission and Occurrence of MTX Resistance

Variable	Outcome		p-value
	Remission n = 25	Resistance n = 5	
Group			0.014*
Intervention	16 (100.0%)	0 (0.0%)	
Control	9 (64.3%)	5 (35.7%)	
Age			0.005*
Mean	31 ± 9	43 ± 4	
Median	32	41	
Range (min–max)	19–46	40–48	
Initial β-hCG level			0.231
Mean	182.807.6 ± 350.287.20	133.735.2 ± 96.696.7	
Median	13.070.0	147.908.0	
Range (min–max)	50.0–1.125.000.0	9.300–273.700.0	

For categorical data, the p-value was calculated based on the Chi-Square test, or the Kolmogorov Smirnov and Exact Fisher tests if the requirements of the Chi-Square were not met. For numerical data, the p-value was generated by an unpaired t-test if the data were normally distributed, and by the Mann-Whitney test if the data were not. *Indicates significance (p < 0.05).

## Discussion

Initial and post-cycle serum β-hCG levels were compared between the intervention and control groups, and were shown to be significantly different, with lower average levels of post-cycle β-hCG levels observed in the intervention group (p < 0.05). The percentage change in post-cycle β-hCG levels in the intervention group (−98.52%) were greater than those in the control group (−77.97%), although this was not statistically significant (p > 0.05).

Vitamins are essential micronutrients that play an important role in physiological processes, including vision, immune response, cell differentiation and proliferation, optimal intracellular communication, and reproduction. Vitamins can be divided into water-soluble (vitamins B and C) and fat soluble (vitamins A, D, E, and K).

Vitamin A is a fat soluble vitamin which is absorbed through a carrier protein (Goncalves et al., 2015). Low levels of vitamin A are thought to be a predisposing factor of trophoblastic cell proliferation in hydatidiform mole (Andrijono et al., 1997; Andrijono et al., 2007; Andrijono and Muhilal, 2010). Two case-control studies demonstrated that the risk of molar pregnancy progressively increased with reduced intake of animal fat and carotene, which are the precursors of vitamin A (Garner et al., 2007). Two other previous studies also showed that vitamin A helps to decrease serum β-hCG levels in patients with low-risk GTT who undergo MTX therapy (Sutanto et al., 2012; Ghasemian et al., 2018). Vitamin A is known to increase trophoblast cell regression, decrease β-hCG levels, and increase the effectiveness of MTX by inducing more malignant cell death than MTX alone (Ghasemian et al., 2018).

Normal levels of vitamin A in the blood vary from 15–60 mcg/dL or 0.52–2.09 μmol/L. Normal vitamin A levels will vary by laboratory, given the different detection methods (Chernecky and Berger, 2012; Chernecky, 2013). Two cohort studies have stated that low levels of retinol are associated with an increased risk of cancer (Wald et al., 1980; Kark et al., 1981).

The incidence of resistance between the intervention groups was significantly lower than that of the control group (p < 0.05). Chemotherapy resistance, whether spontaneous or after therapy, is a common problem (Schimke, 1986), and three mechanisms have been described for the occurrence of MTX resistance: (1) MTX affinity disorder for dihydrofolate-reductase (DHFR); (2) MTX transport interruptions; and (3) overproduction of DHFR (Schimke, 1986). A retrospective study conducted in the United States reviewed medical records of 358 low-risk TTG patients from 1979–2009, and found that 81% of patients had complete remission with initial MTX chemotherapy, while the remaining 19% needed second line therapy or multi-agent chemotherapy with and without surgery. Resistance to initial MTX chemotherapy was associated with an increase in FIGO scores, choriocarcinoma histopathology, pre-chemotherapy hCG, and the presence of metastasis (Chapman-Davis et al., 2012).

In conclusion, in patients with low-risk GTN who received MTX chemotherapy, β-hCG levels and the incidence of chemo resistance were lower in the intervention group than in the control group. Older age could also influence the incidence of chemo resistance in GTN patients, while oral administration of 6000 IU vitamin A could help to reduce β-hCG levels in low-risk GTN patients who receive MTX chemotherapy.
